# 

**Published:** 1874-05-15

**Authors:** 


AMERICAN MEDICAL ASSOCIATION. —Let none of our readers forget that this Association meets this year in Detroit, on Tuesday, June 2d, 1874. We understand that all necessary efforts are being made to have the coming meeting an important and profitable one. The amendments to the Constitution and By-Laws, made last year, providing for a permanent Judicial Counsel, and the presentation of reports or addresses on the different departments of medicine by the Chairmen of Sections, will add very much to the interest and value of the work, both in the General Sessions and Sections. It is to be hoped that a full representation will be present from all sections of the country.

